# Impaired Antiviral Responses to Extracellular Double-Stranded RNA and Cytosolic DNA, but Not to Interferon-α Stimulation, in TRIM56-Deficient Cells

**DOI:** 10.3390/v14010089

**Published:** 2022-01-05

**Authors:** Dang Wang, Ruixue Wang, Kui Li

**Affiliations:** Department of Microbiology, Immunology and Biochemistry, University of Tennessee Health Science Center, Memphis, TN 38163, USA; wangdang511@126.com (D.W.); rwang28@uthsc.edu (R.W.)

**Keywords:** TRIM56, interferon, interferon-stimulated gene, ISGylation, toll-like receptor 3, double-stranded RNA, gene knockout, cyclic GMP–AMP synthase, vesicular stomatitis virus, antiviral

## Abstract

The physiologic function of tripartite motif protein 56 (TRIM56), a ubiquitously expressed E3 ligase classified within the large TRIM protein family, remains elusive. Gene knockdown studies have suggested TRIM56 as a positive regulator of the type I interferon (IFN-I) antiviral response elicited via the Toll-like receptor 3 (TLR3) and cyclic GMP–AMP synthase (cGAS)–stimulator of interferon genes (STING) pathways, which detect and respond to danger signals—extracellular double-stranded (ds) RNA and cytosolic dsDNA, respectively. However, to what extent these pathways depend on TRIM56 in human cells is unclear. In addition, it is debatable whether TRIM56 plays a part in controlling the expression of IFN-stimulated genes (ISGs) resulting from IFN-I based antiviral treatment. In this study, we created HeLa-derived TRIM56 null cell lines by gene editing and used these cell models to comprehensively examine the impact of endogenous TRIM56 on innate antiviral responses. Our results showed that TRIM56 knockout severely undermined the upregulation of ISGs by extracellular dsRNA and that loss of TRIM56 weakened the response to cytosolic dsDNA. ISG induction and ISGylation following IFN-α stimulation, however, were not compromised by TRIM56 deletion. Using a vesicular stomatitis virus-based antiviral bioactivity assay, we demonstrated that IFN-α could efficiently establish an antiviral state in TRIM56 null cells, providing direct evidence that TRIM56 is not required for the general antiviral action of IFN-I. Altogether, these data ascertain the contributions of TRIM56 to TLR3- and cGAS–STING-dependent antiviral pathways in HeLa cells and add to our understanding of the roles this protein plays in innate immunity.

## 1. Introduction

Tripartite motif protein 56 (TRIM56) is a member of the large TRIM protein family of E3 ligases that are involved in a broad array of host processes, including, but not limited to, proliferation, differentiation, development and, recently, immunity [1,2,3,4,5,6,7]. Although TRIM56 is broadly expressed in different human tissues albeit at varying levels [3], little is known about the normal cellular function of this protein. Interestingly, several recent studies have linked TRIM56 to antiviral innate immunity. Specifically, ectopic expression of TRIM56 in cell culture inhibits the propagation of several flaviviruses including bovine viral diarrhea virus, yellow fever, dengue and Zika viruses [3,8,9], human coronavirus OC43 [8], influenza A and B viruses [10], and HIV [11]. A role for TRIM56 in protecting mice against HSV-1 infection has also been suggested [6].

In addition to its reported antiviral activities, TRIM56 has also been implicated in regulating innate immune-signaling pathways that culminate in the induction of type I interferon (IFN-I) response, a hallmark of the intrinsic, immediate defense mechanisms of mammalian hosts against viral infections. In keeping with this, TRIM56 expression *per se* is moderately upregulated by IFN-I [3,5,9], as often is the case with many other innate immune regulators. Gene knockdown experiments in HEK293 [4], HeLa [5], and THP-1 [6] cells have demonstrated that TRIM56 contributes to the cytosolic DNA-sensing pathway, although it is controversial whether the substrate for the TRIM56 E3 ligase is cyclic GMP–AMP synthase (cGAS) [6] or its downstream adaptor, stimulator of interferon genes (STING) [4]. Separately, experiments based on RNA interference (RNAi)-mediated depletion of TRIM56 in HEK293 and Huh7.5 cells reconstituted with Toll-like receptor 3 (TLR3) expression and in HeLa cells harboring a physiologic level of TLR3 had suggested a critical role of TRIM56 in this viral double-stranded (ds) RNA-sensing pathway [5]. Of note, in contrast to the mechanism proposed for the TRIM56 regulation of the cGAS-STING pathway that hinges on the E3 ubiquitin ligase activity, TRIM56 promotes IFN-I and chemokine production via the TLR3 pathway in a non-canonical, E3 ligase-independent fashion. Rather, such capacity correlates with an interaction of TRIM56 with Toll/interleukin-1-receptor-domain-containing adapter-inducing interferon-β (TRIF), the adaptor for TLR3. It remains unclear, however, to what extent these innate immune mechanisms depend on TRIM56 in human cells. Studies in TRIM56 null cells are needed to answer this critical question and to corroborate previous experimental findings.

There also is a discrepancy regarding whether TRIM56 has a role in modulating the induction of IFN-stimulated genes (ISGs), whose products act in concert to execute the antiviral actions of IFNs. In immunoblotting experiments, Shen et al. found that upregulation of representative ISGs by IFN-α was normal in TRIM56 knockdown cells [5]. Kane et al., on the other hand, reported that depletion of TRIM56 attenuated the induction of transcript for a subset of ISGs by IFN-α [11]. The latter group proposed that accentuation of ISG induction by IFN-α is a mechanism by which TRIM56 enhances the antiretroviral activity of IFN-α.

In this study, we set out to create TRIM56-deficient cell lines by CRISPR/Cas9 gene editing, and to address the requirements for this protein in various innate immune pathways in human cells. We report that TRIM56 deletion severely compromises antiviral gene expression induced via the TLR3 signaling pathway and that its loss weakens the response through the cytosolic DNA-sensing pathway. By contrast, our data do not support a significant role, if any, of TRIM56 in regulating ISG induction downstream of the IFN-I receptors or impacting the establishment of a general antiviral state by IFN-I.

## 2. Materials and Methods

### 2.1. Plasmids

The retroviral vector pCX4pur-FH-TRIM56 encoding N-terminally Flag- and HA-tandem tagged human TRIM56 (FH-T56) in the pCX4pur backbone has been described previously [9]. The two all-in-one CRISPR/Cas9 plasmids expressing the Cas9 nuclease, as well as a 20-nucleotide long, single-guide RNA (sgRNA) specifically targeting the exon 3 of human TRIM56 genomic DNA, were provided by Horizon Discovery Group Ltd. (Cambridge, United Kingdom). The target-specific sgRNA sequences were: TRIM56_172121, GGCCAGGAAGTCGCTGCTCA and TRIM56_172123, TGGCAGTAGGTATGCAGGCA, respectively.

### 2.2. Cell Lines

HeLa and Vero cells (both from ATCC, Manassas, Virginia, USA) were maintained in Dulbecco’s modified Eagle medium supplemented with 10% fetal bovine serum (FBS), 100 U/mL penicillin, and 100 μg/mL streptomycin. To create TRIM56 null cell lines by CRISPR/Cas9 gene editing, HeLa cells were co-transfected with TRIM56_172121 and TRIM56_172123 constructs at a 1:1 ratio. Forty-eight hours later, cells were dissociated from the culture plate via trypsin digestion to form single cell suspension and subsequently reseeded onto 96-well plates at a cell density of ~0.5 cells per well. The plates were returned to culture and inspected periodically for wells that contained a single colony. After ~3 weeks, individual cell colonies were expanded and screened for TRIM56 expression, in comparison with parental HeLa cells, by Western blot. Two independent cell lines, clone 17 (referred to as T56-KO#1) and clone 3 (referred to as T56-KO#2), were found to completely lack TRIM56 protein and selected for further analyses.

To stably express Flag- and HA-tandem tagged TRIM56 (FH-T56) in TRIM56 null cells, we transduced T56-KO#1 and T56-KO#2 cells with a replication-incompetent retrovirus packaged from pCX4pur-FH-TRIM56. Following selection in puromycin-containing medium, surviving cell colonies were pooled, designated HeLa-T56-KO#1-FHT56 and HeLa-T56-KO#2-FHT56 cells, respectively, and used for analyses.

### 2.3. Pattern Recognition Receptor Ligands, IFN-α, and VSV-Luc

Poly(I:C), poly(dA:dT), Calf Thymus DNA (C-T DNA), and recombinant human IFN-α 2b were obtained from Sigma (St. Louis, MO, USA). VSV-Luc, a recombinant, firefly luciferase (FLuc)-encoding vesicular stomatitis virus, was a gift from Sean Whelan [12]. VSV-Luc stocks were propagated and titrated by plaque assay on Vero cells.

### 2.4. Stimulation of Cells and Antiviral Activity Assay

Cells were stimulated by various PRR ligands for the indicated time periods to elicit innate immune responses—specifically for this study, ISG expression. To engage the TLR3 pathway, poly(I:C) was added directly into culture medium at a final concentration of 20–50 μg/mL. To activate the cytosolic DNA-sensing pathway, poly(dA:dT) or C-T DNA was transfected into cells at 3 μg per well of a 6-well plate after being complexed with Lipofectamine 2000 at a 1:1 (μg:μL) ratio. To determine ISG induction by IFN-α, cells were incubated with recombinant IFN-α at the indicated concentration for 6 and 12 h, respectively.

To directly gauge the efficacy of IFN-mediated establishment of antiviral state, cells were mock-stimulated or stimulated by IFN-α at indicated concentrations for 16 h, followed by challenge with VSV-Luc at an MOI of 0.1. At 8 h post infection, cells were lysed for firefly luciferase assay as a readout for VSV replication.

### 2.5. Quantitative PCR

Total RNA was extracted from cells following various treatments using TRIzol (Invitrogen) as per the manufacturer’s protocol. Complementary DNA synthesis by reverse transcription and SYBR green-based quantitative PCR (qPCR) were implemented as described previously [13,14]. Briefly, 1 μg of total RNA was programmed for synthesis of cDNA by MMLV reverse transcriptase (Promega) in a 20 μL reaction. The expression of ISG mRNAs, including those for interferon-induced protein with tetratricopeptide repeats 1 (IFIT1), IFIT3, melanoma differentiation-associated protein 5 (MDA5) and 2′-5′-oligoadenylate synthetase 1 (OAS1) in the cDNA samples, was then analyzed by qPCR using gene-specific primers. The primers for IFIT1 [5], IFIT3 [15], and OAS1 [14] have been described. The MDA5 primers were: CATCTGATTGGAGCTGGACA (forward) and TGCCACTGTGGTAGCGATAA (reverse). The relative abundance of each target was normalized to that of 28S rRNA.

### 2.6. Protein Analyses

Cell lysates were prepared in RIPA buffer, quantified for protein concentrations, and subjected to SDS-PAGE and immunoblotting as previously described [9,16]. The following monoclonal (mAb) and polyclonal (pAb) antibodies were used: mouse anti-IFIT3 mAb (Santa Cruz Biotechnology); mouse anti-ISG15 mAb (Santa Cruz Biotechnology); mouse anti-GAPDH mAb (ABclonal Technology); mouse anti-ACTB mAb (ABclonal Technology); rabbit anti-ISG15 pAb (for analysis of ISGylation, a gift from Arthur Haas, Louisiana State University Health Sciences Center); rabbit anti-MDA5 pAb (Proteintech); rabbit anti-IFIT1/ISG56 pAb [16]; and mouse anti-TRIM56 pAb and mAbs (generated by immunizing mice at ABclonal Technology with a recombinant protein antigen comprising the C-terminal 392 aa of human TRIM56 fused to maltose-binding protein and expressed and purified from *E. coli*) [9]. Following incubation with appropriate IRDye-labeled secondary antibodies—goat anti-mouse IgG IRDye^®^ 680RD or goat anti-rabbit IgG IRDye^®^ 800CW (both from LI-COR Biosciences, Lincoln, NE, USA), protein bands were imaged with an Odyssey infrared imaging system (LI-COR Biosciences). To compare target protein expression levels across different samples in immunoblotting, the signal intensity of protein band(s) of interest was determined by Image Studio Lite (LI-COR Biosciences) and normalized to that of a loading control, as indicated.

### 2.7. Statistical Analysis

All results are presented as means ± standard deviations. Statistical differences between two groups were analyzed using a two-tailed Student’s *t*-test (Excel 2016, Microsoft, Redmond, WA, USA). A *p* value of <0.05 was regarded as significant.

## 3. Results

### 3.1. Knockout of TRIM56 Severely Compromises, but Does Not Eliminate Extracellular dsRNA-Induced Antiviral Gene Expression

**Figure 1:**
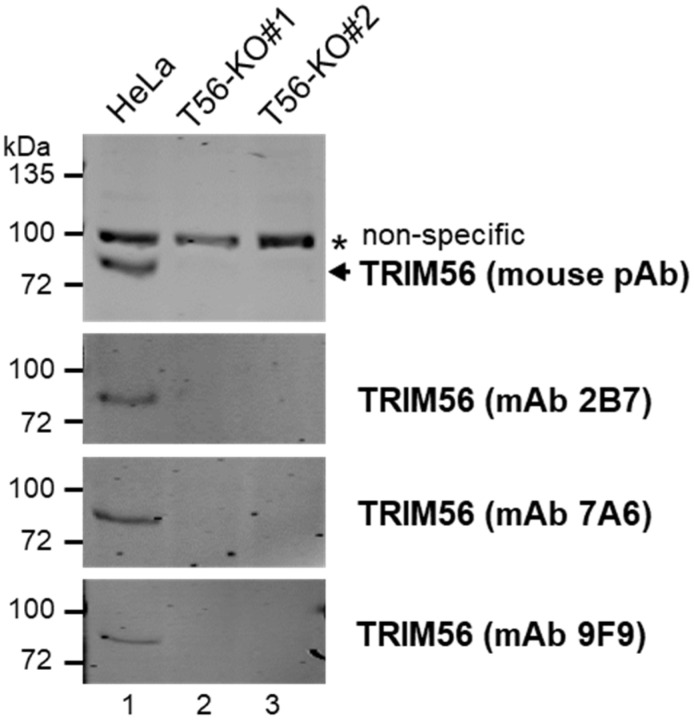
Characterization of HeLa-derived TRIM56 knockout cell lines created by CRISPR/Cas9 gene editing. The expression of endogenous TRIM56 protein in parental HeLa and two HeLa-TRIM56-KO cell lines was probed by immunoblotting using a mouse polyclonal hyperimmune antiserum (pAb) against recombinant TRIM56 (top panel) or the culture supernatant of three independent hybridoma cell lines (2B7, 7A6, and 9F9) secreting anti-TRIM56 monoclonal antibody (mAb) derived from this immunized mouse (three lower panels). A nonspecific band (marked by *) detected by the mouse pAb served as a loading control. The immunoblotting data are representative of five independent experiments.

To clarify the roles of TRIM56 expressed at physiologic levels in innate immune signaling, we performed CRISPR/Cas9-mediated gene editing to eliminate TRIM56 expression in HeLa cells. HeLa was chosen as the founder to create TRIM56 KO cells because this well-characterized cell line (1) has been widely used as a cell culture model to study innate immunity against viral infections, (2) possesses intact dsRNA- and dsDNA-sensing antiviral pathways including those dependent on TLR3 [5] and on cGAS-STING [17,18], and (3) expresses readily detectable, endogenous TRIM56 mRNA and protein [3,5]. Two independent clonal cell lines, designated HeLa-T56-KO#1 and HeLa-T56-KO#2, were found to be devoid of TRIM56 expression by immunoblotting. The absence of TRIM56 protein was corroborated by using several different TRIM56 antibodies, including a mouse polyclonal, hyperimmune serum raised against a recombinant TRIM56 fragment encompassing C-terminal 392 aa of the protein, or using the culture supernatant of several independent hybridoma cell lines derived from this immunized mouse (Figure 1). As a control, parental HeLa cells were found to harbor abundant TRIM56 protein, confirming the sensitivity of our immunoblotting conditions.

**Figure 2:**
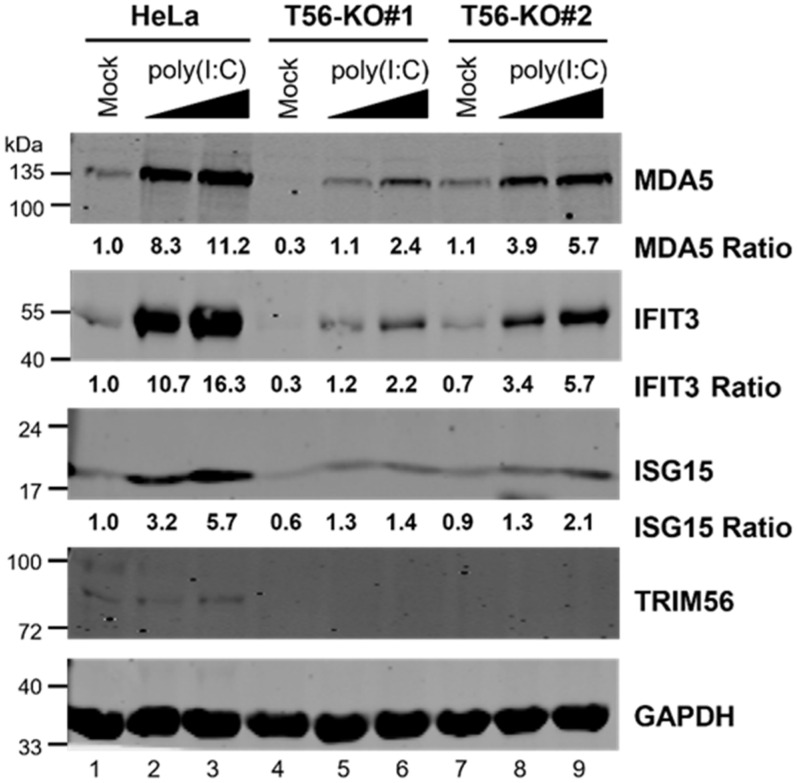
The induction of ISGs by extracellular poly(I:C) is severely compromised in HeLa-TRIM56-KO cells. Parental HeLa and two HeLa TRIM56 knockout cell lines (T56-KO#1 and T56-KO#2) were mock-stimulated or stimulated by increasing concentrations of poly(I:C) (20 μg/mL and 50 μg/mL, respectively) added directly to culture medium. Sixteen hours later, cells were lysed for immunoblotting detection of MDA5, IFIT3, ISG15, TRIM56, and GAPDH (loading control) proteins. The relative expression of indicated ISG protein in each sample is presented as a ratio relative to that of mock-stimulated HeLa cells (lane 1) after normalization to endogenous GAPDH protein expression. The immunoblotting data are representative of three independent experiments.

Next, we investigated the effect of TRIM56 deficiency on TLR3 signaling by comparing HeLa-T56-KO#1 and -KO#2 with parental HeLa cells for ISG induction following poly(I:C) stimulation via the extracellular route, which engages specifically the TLR3 pathway [19]. Immunoblotting experiments demonstrated that poly(I:C) robustly upregulated the expression of three representative antiviral ISGs (i.e., ISG15, IFIT3, and MDA5) in a dose-dependent fashion, in control HeLa cells (Figure 2, compare lanes 3 and 2 vs. lane 1). In contrast, these responses were severely compromised in both HeLa-T56-KO lines (lanes 4–6 for KO#1 and lanes 7–9 for KO#2). The impairment was especially severe in KO#1 cells. These data corroborate our previous finding based on RNAi knockdown experiments demonstrating that TRIM56 is a critical component of and facilitates signaling through the TLR3 pathway [5]. Additionally, they reveal that a minor fraction of extracellular dsRNA-induced antiviral gene expression is TRIM56-independent because a residual, dose-dependent response to poly(I:C) was detected in both T56-KO cell lines (Figure 2, lanes 4–6 and 7–9). Notably, resembling cell lines with stable TRIM56 knockdown [5], T56-KO#1 had substantially lower levels of basal expression for all 3 ISG proteins than control HeLa (Figure 2, compare lane 4 vs. lane 1). However, this was not case with T56-KO#2 (compare lane 7 vs. lane 1). The reason for this difference is unclear but could reflect clonal variations. Nevertheless, both TRIM56 KO cell lines exhibited diminished ISG induction following stimulation by poly(I:C) administered directly in culture medium, confirming a pivotal role of TRIM56 in antiviral gene expression elicited via the TLR3 pathway.

### 3.2. Reconstitution of TRIM56 Expression in HeLa T56-KO Cell Lines Reverses the Impaired TLR3 Response Phenotype

**Figure 3:**
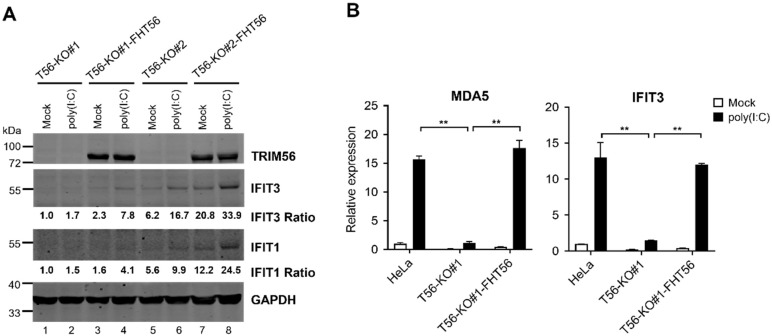
Reconstitution of TRIM56 expression reverses the impaired TLR3 response phenotype in HeLa-T56-KO cell lines. (**A**) HeLa-T56-KO#1 and -KO#2 cells with (T56-KO#1-FHT56 and T56-KO#2-FHT56) and without stable reconstitution of Flag-HA-tagged TRIM56 (FHT56) were mock-stimulated or stimulated by 40 μg/mL of poly(I:C) that was added directly to culture medium. Eight hours later, cells were lysed for immunoblotting of TRIM56, IFIT3, IFIT1, and GAPDH (loading control) expression. The relative expression of indicated ISG protein in each sample is presented as a ratio relative to that of mock-stimulated HeLa-T56-KO#1 cells (lane 1) after normalization to endogenous GAPDH protein expression. (**B**) Impact of TRIM56 deletion on the induction of ISG mRNAs by extracellular poly(I:C). Parental HeLa, T56-KO#1, and T56-KO#1 reconstituted with TRIM56 expression (T56-KO#1-FHT56) were mock-treated or stimulated by 40 μg/mL of poly(I:C). Eight hours later, cells were harvested for total RNA exaction and qPCR analysis of the abundance of MDA5 and IFIT3 mRNAs (relative to that of mock-treated HeLa cells after normalization to endogenous 28S mRNA). ** denotes *p* < 0.01. The qPCR data are representative of two independent experiments.

We considered the remote chance that the profound impairment in TLR3 response of the two TRIM56 null cell lines was intrinsic to the cell clones selected, independent of TRIM56 deletion. To exclude this possibility, we reconstituted TRIM56 expression in HeLa-T56-KO#1 and -KO#2 cells by retroviral gene transfer of FLAG- and HA-tandem tagged human TRIM56 (FH-T56) and compared the reconstituted cells with their untransduced counterparts for ISG induction by extracellular poly(I:C). We chose an early time point (8 h) post stimulation, when the response was still in its climbing phase, for immunoblotting detection of two representative ISGs, IFIT3 and IFIT1. This strategy avoided the plateau phase of the TLR3 response, ensuring that any difference between T56-KO and -reconstituted cells would be captured. Consistent with our earlier data, there was little induction of either ISG by poly(I:C) in T56-KO#1 (Figure 3A, compare lane 2 vs. lane 1) or T56-KO#2 cells (compare lane 6 vs. lane 5). In contrast, the poly(I:C) upregulation of both IFIT proteins was evident in cells stably transduced for FH-T56 expression, irrespective of clonal origin (compare lane 4 vs. lane 3 for KO#1-FH-T56 and lane 8 vs. lane 7 for KO#2-FH-T56). To further confirm this result, we conducted qPCR quantifying the poly(I:C) induction of transcript for MDA5 and IFIT3 in parental HeLa, T56-KO#1, and T56-KO#1-FH-T56 cells (Figure 3B). Consistent with the Figure 3A immunoblotting data, the upregulation of both ISG mRNAs following poly(I:C) stimulation was significantly inhibited in the TRIM56-deficient T56-KO#1 cells, compared with parental HeLa cells. The diminished response to poly(I:C) in TRIM56 null cells, however, was reversed after FH-T56 was reconstituted (T56-KO#1-FH-T56). Collectively, these data validate that the diminished TLR3 response in T56-KO cell lines is indeed a consequence of TRIM56 loss.

### 3.3. TRIM56 Deficiency Is Associated with Reduced ISG Response to Cytosolic dsDNA

**Figure 4:**
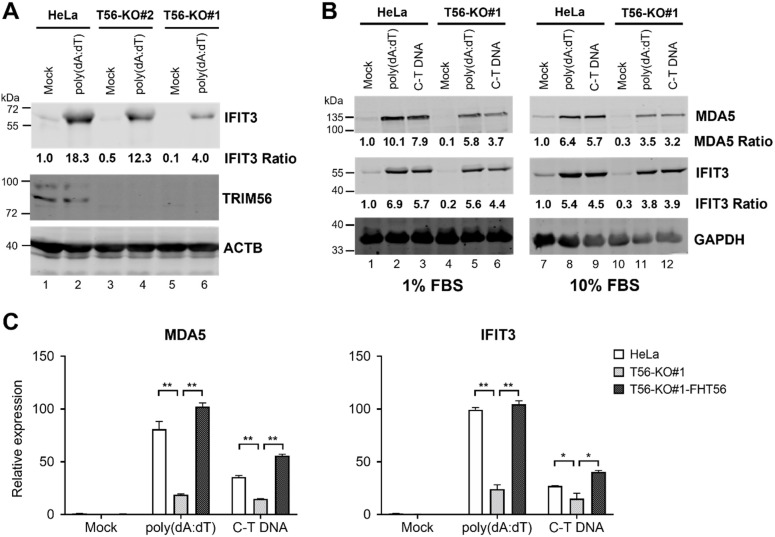
TRIM56 deficiency is associated with reduced ISG response to cytosolic dsDNA. (**A**) Parental HeLa and HeLa cell lines knockout for TRIM56 (T56-KO#1 and T56-KO#2) were mock-transfected or transfected with poly(dA:dT) at 3 μg per well of 6-well plate. Eight hours later, cells were lysed for immunoblotting of IFIT3, TRIM56, and ACTB (loading control) expression. Data are representative of three independent experiments. (**B**) Parental HeLa and HeLa cell line knockout for TRIM56 (T56-KO#1) were seeded onto 6-well plates and cultured in 10% FBS-containing medium overnight. One hour prior to transfection, culture medium was replaced with that supplemented with different FBS concentrations (1% or 10% FBS, respectively). Cells were then mock-stimulated or transfected with poly(dA:dT) or calf thymus genomic DNA (C-T DNA) at 3 μg per well. Eight hours later, cells were lysed for immunoblotting of MDA5, IFIT3, and GAPDH (loading control) expression. The relative expression of indicated ISG protein in each sample is presented as a ratio relative to that of mock-stimulated parental HeLa cells after normalization to endogenous ACTB (**A**) or GAPDH (**B**) protein expression. (**C**) Impact of TRIM56 deletion on the induction of ISG mRNAs by cytosolic dsDNA. Parental HeLa, T56-KO#1, and T56-KO#1-FHT56 cells cultured in 1% FBS-containing medium as described in (**A**) were mock-treated or transfected with poly(dA:dT) or C-T DNA. Eight hours later, cells were harvested for total RNA exaction and qPCR analysis of the abundance of MDA5 and IFIT3 mRNAs (relative to that of mock-treated HeLa cells after normalization to endogenous 28S mRNA). * and ** denote *p* < 0.05 and *p* < 0.01, respectively. The qPCR data are representative of two independent experiments.

Several previous studies have shown that RNAi-mediated knockdown of TRIM56 undermines the IFN response triggered by cytosolic delivery of DNA [4,5,6], which engages the cGAS-STING pathway. We determined whether this was the case in HeLa cells completely lacking TRIM56. To this end, we stimulated HeLa-T56-KO#1 and -KO#2 cells, in comparison with parental HeLa cells, with poly(dA:dT), a dsDNA surrogate, by lipofectamine-mediated transfection. Immunoblotting data revealed that upregulation of IFIT3 was profoundly curtailed in T56-KO#1 cells (Figure 4A, compare lane 6 vs. lane 2), while reduced in T56-KO#2 cells albeit to a less extent (compare lane 4 vs. lane 2). Apart from primarily triggering cGAS/STING-dependent IFN production, poly(dA:dT) can elicit retinoic-inducible gene I (RIG-I)-dependent signaling through an RNA polymerase III-transcribed RNA intermediate [20,21]. Given that ISG response via the RIG-I/MDA5 pathway does not require TRIM56 [5], we sought to verify the effect of TRIM56 deletion using C-T DNA, a more specific ligand for the cGAS/STING pathway. As shown in Figure 4B, immunoblotting data showed that induction of ISGs (IFIT3 and MDA5) by C-T DNA was also substantially weakened in TRIM56 null cells, compared with control HeLa cells. Notably, similar results were obtained when cells were cultured in 10% or 1% FBS, the latter condition having been suggested to render HeLa cells more responsive to cytosolic DNA stimulation [22]. Moreover, qPCR analyses showed that the upregulation of MDA5 and IFIT3 mRNAs by cytosolic poly(dA:dT) or C-T DNA was significantly lower in TRIM56 null cells (T56-KO#1) than in parental HeLa, but was restored in TRIM56 null cells that had been reconstituted with FH-T56 (T56-KO#1-FH-T56) (Figure 4C). These results corroborate the immunoblotting data (Figure 4A,B). Further, they illustrate that the impaired ISG response via the cGAS/STING pathway in TRIM56 KO cells resulted from deletion of the gene. In aggregate, data from these experiments demonstrate that TRIM56 participates in cellular antiviral responses elicited by cytosolic DNA, although its impact seems to vary depending on cellular context (see discussion below).

### 3.4. TRIM56 Deletion Does Not Impair ISG Induction by IFN-α

**Figure 5:**
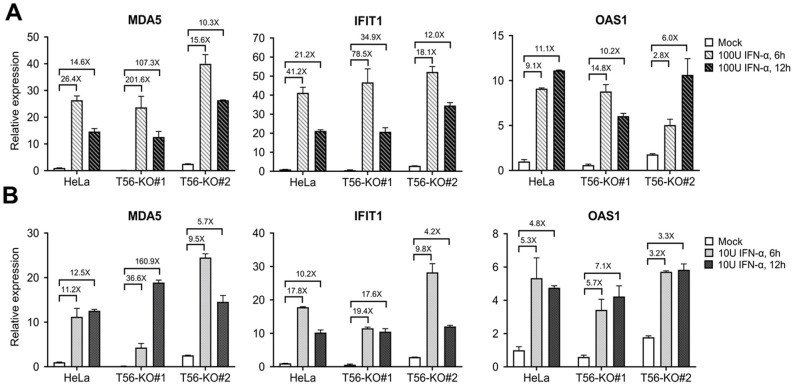
Impact of TRIM56 deletion on the induction of ISG mRNAs by IFN-α. Parental HeLa and HeLa cell lines knockout for TRIM56 (T56-KO#1 and T56-KO#2) were mock-treated or stimulated with 100 U/mL (**A**) or 10 U/mL (**B**) of IFN-α. Six and twelve hours later, cells were harvested for RNA exaction and qPCR analysis of the abundance of MDA5, IFIT1, and OAS1 mRNAs (relative to that of mock-treated HeLa cells after normalization to endogenous 28S mRNA). Data shown are representative of three independent experiments.

Next, we determined whether cells with and without endogenous TRIM56 expression differed in their response to IFN-I. HeLa and the two T56-KO cell lines were stimulated by high (100 U/mL) and low (10 U/mL) concentrations of IFN-α for 6 and 12 h, respectively, and subsequently lysed for RNA extraction and RT-qPCR analysis of the expression for three representative ISGs, MDA5, IFIT1, and OAS1. As shown in Figure 5A, following high IFN concentration treatment, all three ISGs reached their peak levels of induction at 6 h in parental HeLa cells. Thereafter, expression of MDA5 and IFIT1 receded by ~40–50%, while OAS1 transcript remained largely steady, at 12 h post stimulation. Although basal levels of these ISGs were all lower in T56-KO#1 cells than in HeLa to varying degrees, their expression pattern after IFN stimulation followed a similar track and all three ISG mRNAs reached peak levels that were on par with HeLa. When we compared fold change in ISG expression based on each cell line’s own basal level, T56-KO#1 cells actually responded to IFN stimulation more robustly than HeLa. In comparison, basal expression of all three ISGs were slightly higher in T56-KO#2 cells than in HeLa. Nonetheless, T56-KO#2 cells also responded to IFN treatment efficiently, with the expression of these ISGs peaking at higher or comparable levels than HeLa. Therefore, despite some variations in basal ISG expression in the T56-null cell lines, TRIM56 deficiency does not negatively affect ISG induction by IFN-α. The same could be said when cells were stimulated by low concentration of IFN-α (Figure 5B).

### 3.5. ISGylation Takes Place Efficiently in the Absence of TRIM56

**Figure 6:**
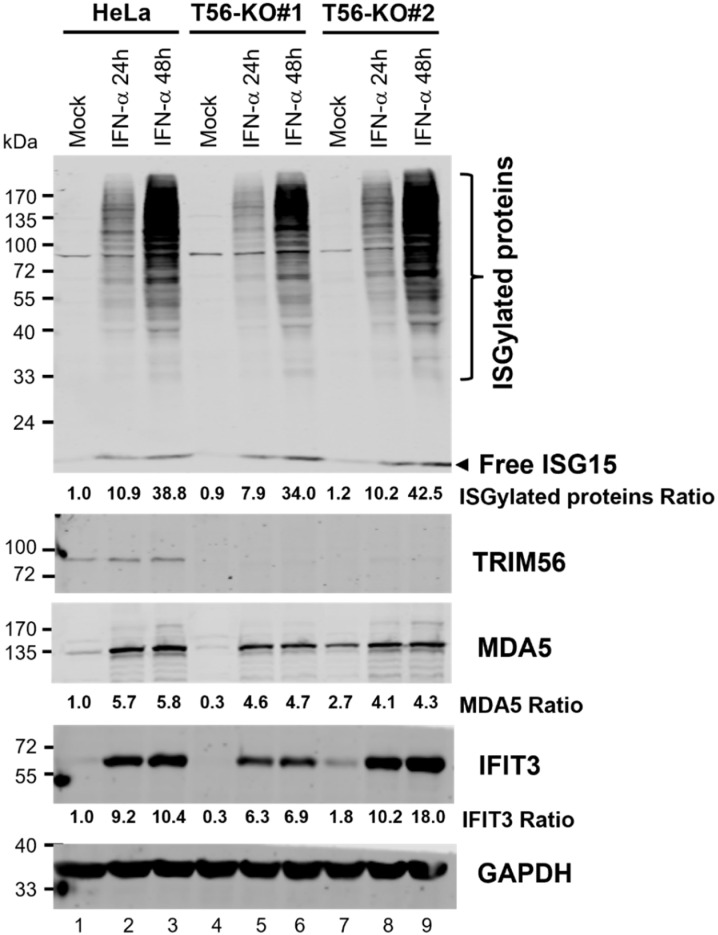
Impact of TRIM56 deficiency on ISGylation and ISG proteins expression following IFN-α stimulation. Parental HeLa and HeLa cell lines knockout for TRIM56 (T56-KO#1 and T56-KO#2) were mock-treated or stimulated with IFN-α (400 U/mL) for 24 h or 48 h. Cell lysates were analyzed by Western blot to probe the expression of free ISG15 and ISG15-conjugated protein products TRIM56, MDA5, IFIT3, and GAPDH (loading control). The relative expression of indicated target protein(s) in each sample is presented as a ratio relative to that of mock-stimulated HeLa cells (lane 1) after normalization to endogenous GAPDH protein expression.

ISGylation, characterized by a ubiquitination-like process covalently linking ISG15 to a subset of protein targets, contributes to IFN-mediated antiviral protection against some, but not all, viruses [23]. One member of the TRIM family of E3 ligases, TRIM25, has been implicated in the ISGylation process by acting as an E3 ligase [24]. It is not known, however, whether other TRIM proteins also play a part in regulating ISGylation. We thus determined whether the physiological level of TRIM56 had any impact on global ISGylation. HeLa and the two T56-KO cell lines were stimulated by IFN-α for 24 and 48 h, respectively, or left unstimulated, followed by immunoblotting using a rabbit anti-ISG15 polyclonal antibody that could detect both free ISG15 and ISGylated cellular proteins. As controls for the effectiveness of IFN stimulation, we also examined the upregulation of two other ISGs, MDA5 and IFIT3. As shown in Figure 6, the expression of free ISG15 protein was strongly induced by IFN-α at 24 h and a slight, further uptick was observed at 48 h post-stimulation. This occurred in control HeLa cells as well as the two TRIM56 null cell lines. Robust induction of MDA5 and IFIT3 was also observed in all three cell lines, although the expression of these two ISG proteins plateaued at 24 h. These immunoblotting data agree with our earlier mRNA data (Figure 5) that suggested ISG induction by IFN-α is not negatively impacted by TRIM56 deletion. Again, we observed that basal expression of MDA5 and IFIT3 proteins was lower in HeLa-T56-KO#1 (but not HeLa-T56-KO#2) than in control HeLa cells (Figure 6, compare lanes 4 vs. 1 and lanes 7 vs. 1, respectively). When it comes to global protein ISGylation, immunoblotting data showed that control HeLa and HeLa-T56-KO#2 cells responded to IFN stimulation at comparable efficiency, with both harboring a readily detectable smear of ISG15-conjugated protein products at 24 h that exhibited a further robust increase in abundance at 48 h (Figure 6, compare lanes 2 and 3 vs. 1 and lanes 8 and 9 vs. 7, respectively). In comparison, IFN-stimulated HeLa-T56-KO#1 cells also exhibited a robust ISGylation response (lanes 4–6) that followed a similar kinetics of induction, although the overall signal intensity was slightly less at each time point. Thus, despite some clonal differences, loss of TRIM56 has a negligible effect on ISGylation.

### 3.6. TRIM56 Is Not Required for the Establishment of an Antiviral State by IFN-α

**Figure 7:**
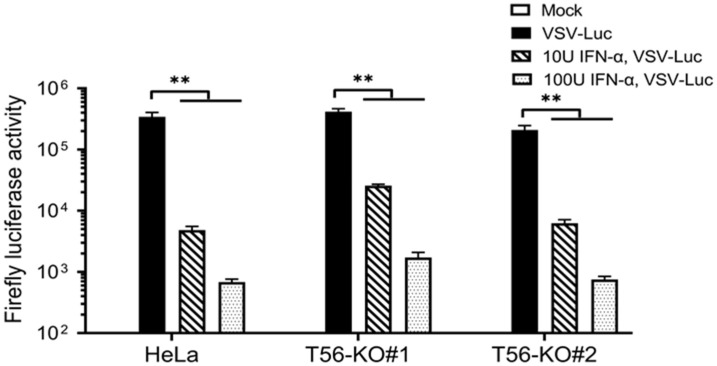
TRIM56 is not required for IFN-α to establish an antiviral state. Parental HeLa and HeLa cell lines knockout for TRIM56 (T56-KO#1 and T56-KO#2) were mock-stimulated or stimulated with IFN-α at 10 U/mL and 100 U/mL, respectively. Sixteen hours later, cells were mock-infected or infected with VSV-Luc (MOI = 0.1) for 8 h before being lysed for firefly luciferase activity assay. Data are representative of four independent experiments. ** denotes *p* < 0.01.

To directly gauge the overall impact, if any, that TRIM56 may have on IFN-mediated establishment of an antiviral state, we conducted VSV challenge experiments in cells with and without prior IFN-α stimulation. VSV is a negative-strand RNA virus highly sensitive to the antiviral action of IFNs. As such, it has been widely used as a tool virus for IFN bioactivity assays. We took advantage of VSV-Luc, a recombinant VSV whose replication can be conveniently and quantitatively monitored by measuring activities of the firefly luciferase reporter the virus encodes, in infected cells. HeLa, HeLa-T56-KO#1, and -KO#2 cells were pre-incubated with low (10 U/mL) and high (100 U/mL) concentrations of IFN-α for 16 h, respectively, or left unstimulated, followed by infection with VSV-Luc (MOI = 0.1) for 8 h. As shown in Figure 7, high levels of viral replication were observed in all three cell lines without IFN pretreatment, with T56-KO#1 cells supporting slightly higher and T56-KO#2 cells slightly lower VSV-Luc replication, than control HeLa cells. This result was correlated with the basal ISG expression status among these cells (Figure 5 and Figure 6). IFN pretreatment at either concentration greatly reduced VSV-Luc replication in all three cell lines, with groups receiving the higher concentration of IFN-α exhibiting a more pronounced antiviral effect (Figure 7). Specifically, viral replication was curtailed by 98.6% and 99.8%, respectively, in control HeLa cells pretreated with 10 and 100 U/mL of IFN-α. In comparison, the numbers (% reduction in VSV-Luc replication by prior incubation with low and high concentrations of IFN-α) were 93.8% and 99.6% for HeLa-T56-KO#1 cells and 97.0% and 99.6% for HeLa-T56-KO#2 cells, respectively. Notably, although low concentration of IFN-α appeared to be marginally less effective in T56-KO#1 cells than in control HeLa (93.8% vs. 98.6%), it was nearly as effective in T56-KO#2 cells (97.0% vs. 98.6% in HeLa). We conclude from these experiments that loss of TRIM56 has a negligible impact on IFN-mediated establishment of a general antiviral state.

## 4. Discussion

In this study, we have created two independent TRIM56 null cell lines from HeLa cells by CRISPR/Cas9 gene editing. Using these TRIM56-deficient cell models and their parental wild-type counterpart, we systematically investigated the influence of physiologic level of TRIM56 on innate antiviral responses, which TRIM56 has been suggested to regulate by several recent studies based on gene knockdown experiments. As discussed below, data described herein help clarify and add to our understanding of the various roles that TRIM56 plays in host intrinsic immune mechanisms fending off viruses.

**Figure 8:**
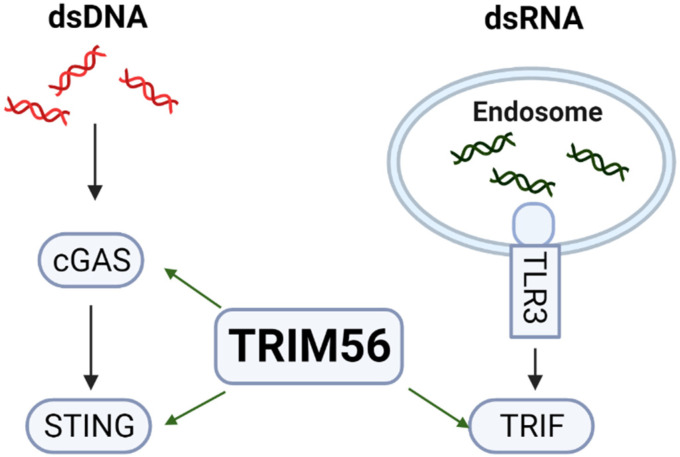
TRIM56 positively regulates pattern recognition receptor pathways elicited by cytosolic dsDNA and extracellular dsRNA via cGAS-STING and TLR3-TRIF, respectively.

In both TRIM56 knockout cell lines, the induction of multiple ISGs following exposure to extracellular dsRNA, which is predominantly mediated via the endocytic TLR3-TRIF pathway [19], was profoundly undercut (Figure 2). This phenotype could be reversed by reconstituting TRIM56 expression (Figure 3), confirming the effect observed was indeed due to loss of TRIM56 and not a consequence of “off-target” associated with CRIPSR/Cas9 gene editing. These data validate the conclusion drawn by Shen et al. [5] that TRIM56 is a critical molecule that facilitates innate antiviral signaling through the TLR3 pathway (Figure 8). Additionally, they demonstrate that a small fraction of the response to extracellular dsRNA is TRIM56-independent, as evidenced by residual ISGs induction in TRIM56 knockout cell lines (Figure 2). Further studies are needed to determine whether this minor portion of innate defense is also TLR3-mediated or elicited via RIG-I/MDA5, the latter constituting a dsRNA-sensing pathway for which TRIM56 is dispensable [5]. Although RIG-I and MDA5 mainly operate in the cytoplasm, each has been reported to contribute, albeit to a minor extent compared with TLR3, to ISG induction by naked poly(I:C) added to culture medium [19,25]. Perhaps these sensors capture traces of dsRNAs that have escaped the endolysosomes after their endocytic uptake or have accessed the cytosol via an entry route independent of endocytosis.

We also observed impaired ISG expression in TRIM56 null cells following stimulation by cytosolic dsDNA (Figure 4), which is in line with previous reports that TRIM56 positively regulates cGAS-STING signaling [4,5,6] (Figure 8). However, ISG expression triggered via this pathway appears to be less dependent on TRIM56 than that via the TLR3 pathway—we observed a substantially decreased response to transfected poly(dA:dT) in T56-KO#1 cells but in T56-KO#2 the effect was moderate (Figure 4). This was not a total surprise. At least one other TRIM protein, TRIM32, functions in a redundant role in activating cGAS-STING signaling [22]. TRIM32, however, does not facilitate but, rather, inhibits TLR3 signaling [26]. It will be interesting to determine in future studies if TRIM32 (or an as-yet-unknown co-factor for TRIM32) is differentially regulated between the two TRIM56 knockout cell lines. Such a scenario may underlie the differing extent to which cytosolic dsDNA-induced antiviral gene expression is dependent on TRIM56 observed with TRIM56 KO clonal cells.

The upregulation of ISGs with antiviral activities is an important mechanism by which IFNs hinder viral replication in infected cells and protect uninfected surrounding cells [27]. In an earlier study, Shen et al. found that although basal expression of ISG15 and IFIT1 proteins were lower in two HeLa clonal cell lines with stable knockdown of TRIM56 than in parental control cells, the induction of both ISGs by IFN-α was not impaired [5]. Kane et al. later reported that depletion of TRIM56 from MT4, a T cell line harboring the human T cell lymphotropic virus-I, was associated with attenuated upregulation of a subset of ISG mRNAs [11]. These authors inferred that TRIM56 augments the antiretroviral activity of IFN-α by enhancing cellular responsiveness to this antiviral cytokine [11]. In this study, we interrogated two HeLa-TRIM56-KO cell lines and their parental counterpart for ISG mRNA and protein expression before and after IFN-α stimulation. We did not find a consistent pattern as to ISG expression level under unstimulated condition, suggesting clonal variations are likely responsible for the difference in basal ISG expression. Despite this, both TRIM56 null cell lines were able to mount a robust ISG response to IFN-α stimulation, at efficiencies (as judged by fold induction) that are comparable, if not better, with control HeLa (Figure 5 and Figure 6). The status of TRIM56 expression was not found to affect ISGylation, a ubiquitin-like protein modification critical for IFN inhibition of some specific viruses, either (Figure 6). Thus, although we only examined a small number of ISGs as representatives, our data do not support that TRIM56 has a significant impact on antiviral responses downstream of the IFN-I receptors, once IFN is synthesized and secreted. VSV challenge experiments in cells pretreated with IFN-α (Figure 7) reinforced this notion and lent direct evidence that a general antiviral state could be efficiently established in cells devoid of TRIM56. We note, however, that these observations were made in HeLa cells of epithelial origin; the possibility cannot be ruled out that TRIM56 may contribute, to some extent, to the expression of subsets of ISGs in cell types of other tissue origins, such as T cells as suggested by Kane et al. in the study conducted in MT4 cells. Along the latter line, TRIM56 is a cytoplasmic protein [3] and not known to be associated with any transcriptional activity in the nucleus. Given that TRIM56 has RNA-binding activity [9], perhaps in some cell types and under specific circumstances, TRIM56 could form a complex with certain cellular mRNAs such as those of specific ISGs, regulating their stability, turnover, or translation. This potential regulation, if indeed in place, is not likely to affect the general antiviral state induced by IFN therapy but may, conceivably, impact the antiviral effect against specific viruses. To address this outstanding, yet relevant, question, future studies in TRIM56-deficient animal models are warranted.

## Data Availability

The authors confirm that the data supporting the findings of this study are available within the article.
